# Potential miRNA involvement in the anti-adipogenic effect of resveratrol and its metabolites

**DOI:** 10.1371/journal.pone.0184875

**Published:** 2017-09-27

**Authors:** Itziar Eseberri, Arrate Lasa, Jonatan Miranda, Ana Gracia, Maria P. Portillo

**Affiliations:** 1 Nutrition and Obesity group, Department of Nutrition and Food Science, University of Basque Country (UPV/EHU) and Lucio Lascaray Research Center, Vitoria, Spain; 2 Centro de Investigación Biomédica en Fisiopatología de la Obesidad y Nutrición (CIBERobn), Instituto de Salud Carlos III, Madrid, Spain; INIA, SPAIN

## Abstract

**Objective:**

Scientific research is constantly striving to find molecules which are effective against excessive body fat and its associated complications. Taking into account the beneficial effects that resveratrol exerts on other pathologies through miRNA, the aim of the present work was to analyze the possible involvement of miRNAs in the regulation of adipogenic transcription factors peroxisome proliferator-activated receptor *γ (ppa*r*γ)*, CCAAT enhancer-binding proteins *α* and *β* (*cebpβ* and *cebpα)* induced by resveratrol and its metabolites.

**Methods:**

3T3-L1 maturing pre-adipocytes were treated during differentiation with 25 μM of *trans*-resveratrol (RSV), *trans*-resveratrol-3-O-sulfate (3S), *trans*-resveratrol-3'-O-glucuronide (3G) and *trans*-resveratrol-4'-O-glucuronide (4G). After computational prediction and bibliographic search of miRNAs targeting *ppa*r*γ*, *cebpβ* and *cebpα*, the expression of microRNA-130b-3p (miR-130b-3p), microRNA-155-5p (miR-155-5p), microRNA-27b-3p (miR-27b-3p), microRNA-31-5p (miR-31-5p), microRNA-326-3p (miR-326-3p), microRNA-27a-3p (miR-27a-3p), microRNA-144-3p (miR-144-3p), microRNA-205-5p (miR-205-5p) and microRNA-224-3p (miR-224-3p) was analyzed. Moreover, other adipogenic mediators such as sterol regulatory element binding transcription factor 1 (*srebf1*), krüppel-like factor 5 (*klf5*), liver x receptor α (*lxrα*) and cAMP responding element binding protein 1 (*creb1*), were measured by Real Time RT-PCR. As a confirmatory assay, cells treated with RSV were transfected with anti-miR-155 in order to measure *cebpβ* gene and protein expressions.

**Results:**

Of the miRNAs analyzed only miR-155 was modified after resveratrol and glucuronide metabolite treatment. In transfected cells with anti-miR-155, RSV did not reduce *cebpβ* gene and protein expression. 3S decreased gene expression of *creb1*, *klf5*, *srebf1* and *lxrα*.

**Conclusions:**

While RSV and glucuronide metabolites exert their inhibitory effect on adipogenesis through miR-155 up-regulation, the anti-adipogenic effect of 3S is not mediated via miRNAs.

## Introduction

Obesity is a genuinely serious real health problem. In 2014 about 13% of the world’s adult population worldwide (11% of men and 15% of women) suffered from obesity [1]. In addition, this pathology induces a great number of co-morbilities, such as type 2 diabetes, dyslipidemia, hypertension and cancer among others. Consequently, direct and indirect costs associated with these common medical conditions have charted a steady rise in obesity costs over the years, as the epidemic has grown.

Adipose tissue growth in obesity can be mediated by hypertrophy, which is to say an increase in adipocyte size and/or hyperplasia, that is an increase in adipocyte number. When hyperplasia takes place there is a stimulation of pre-adipocyte proliferation and further differentiation. The above process, which promotes pre-adipocyte differentiation into mature adipocytes (2) plays a crucial role in the development of obesity and needs to be highly controlled. It is well established that in the long term continued energy overloading can increase this process, mainly in young subjects [2]. Although several molecular aspects of adipogenesis are still unknown, peroxisome proliferator-activated receptor *γ* (*pparγ*) has been identified as the master coordinator of adipocyte differentiation[3]. The control that *pparγ* exerts over pre-adipocytes for them to reach adipocyte functionally needs the expression of other important genes both at the early and at the latter stages of adipocyte differentiation, such as CCAAT enhancer-binding proteins *α* and *β* (*cebpβ* and *cebpα)* respectively [4, 5].

These adipogenic genes are regulated by different mechanisms, microRNAs (MiRNAs) among others. MiRNAs are small non-coding RNAs about 19–23 nucleotides in length that have emerged as important regulators of gene expression [6]. They act by base paring with their target mRNA, which leads to mRNA degradation or translation repression [7, 8]. More than 2500 miRNAs have been described in humans to date [9]. Some of them are involved in numerous physiological and pathological processes, such as energy homeostasis [10], sugar and lipid metabolism [11, 12] and tumorigenesis [13]. As far as adipose tissue is concerned, several studies have concluded that some miRNAs can regulate adipogenesis by targeting genes that regulate this process [14–16].

Scientific research is constantly being undertaken with the aim of finding new molecules, either drugs or food components, which are effective in preventing excess accumulation of body fat and associated complications. This is the case of *trans*-resveratrol (3,4,5-trihydroxystilbene, RSV), a polyphenol with a stilbene structure that consists of two phenolic rings held together by a double styrene bond. This compound is naturally present in various plants, including grapes, berries and peanuts and is produced in response to stress, as a defence mechanism against fungal, viral, bacterial infections and damage from exposure to ultraviolet radiation [17]. Most RSV undergoes rapid and extensive metabolism into enterocytes, before entering blood. Furthermore, it undergoes rapid first-pass metabolism in the liver [17]. Consequently, RSV bioavailability is very low and only a small proportion reaches plasma. The concentrations of glucuronide and sulfate metabolites are relatively higher [18–20]. The proportions of glucuronide and sulfate metabolites depend on the tissue [21] and the species [22]. RSV, which shows antioxidant and antiinflammatory properties, is effective in the prevention of several diseases including cardiovascular diseases, diabetes, cancer and recently, obesity. With regard to obesity, a general consensus concerning the body-fat lowering effect of resveratrol in mice and rats exists [23, 24]. This effect is mainly mediated by a reduction in adipogenesis and lipogenesis and by an increase in energy expenditure, lipolysis and fatty acid oxidation in liver and skeletal muscle [24].

Given the above relating to RSV metabolism, an important question is whether RSV metabolites are active molecules. In a previous study we described how RSV, as well as certain metabolites (*trans*-resveratrol-3-O-sulfate -3S-, *trans*-resveratrol-3'-O-glucuronide -3G- and *trans*-resveratrol-4'-O-glucuronide -4G-) were able to modify the expression of genes related to the adipogenic process [25]. While all of them (RSV, 3G, 4G and 3S) reduced *cebpβ* mRNA levels, only the sulfate metabolite reduced *cebpα* and *pparγ* gene expression.

In this scenario and taking into account that the beneficial effects of RSV on other pathologies, such as cancer and diabetes, are mediated by miRNA [26, 27], the present study focuses on the possible involvement of different miRNAs in the changes induced by RSV and its metabolites in adipogenic transcription factors *ppa*r*γ*, *cebpβ* and *cebpα*, a process which has not been analyzed to date. For this purpose, a well-defined pre-adipocyte model, 3T3-L1 murine adipocytes, was used.

## Material and methods

### Experimental design and cell treatment

The experimental design for 3T3-L1 maturing pre-adipocyte was previously described (25). Briefly, cells grown in 6-well plates were incubated with either 0.1% ethanol (95%) (control group) or with RSV, 3G, 4G or 3S, all of them provided by Bertin Pharma (Montigny le Bretonneux, France), at 25 μM (diluted in 95% ethanol) during the adipogenic phase from day 0 to day 8 of differentiation. The medium was changed every two days. On day 8, supernatant was removed and cells were used for triacylglycerol determination and RNA extraction. Each experiment was performed 3 times.

### MiRNAs selection

For miRNAs selection as potential regulators of *cebpβ*, *cebpα*, *pparγ*, two criteria were established: a) to be validated or predicted by five algorithms (miRanda, miRDB, miRWalk, RNA22 and Targetscan algorithms) in miRWalk 2.0. database [28] and b) to be reported in Pubmed search using “miR + adipogenesis” terms (Table 1).

**Table 1:** miRNAs whose target genes have been predicted or validated by means of the miRWalk 2.0 or reported in the literature.

miRNA	Validated target genes	Predicted target genes (5 algorithms)	Literature Mir+adipogenesis
mmu-miR-31-5p		*cebpα*	[29, 30]
mmu-miR-101a-3p	*cebpα*		-
mmu-miR-101b-3p	*cebpα*		-
mmu-miR-124-3p	*cebpα*	*cebpα*	-
mmu-miR-130b-3p		*pparγ*	[15, 31–33]
mmu-miR-144-3p	*cebpα*		[34]
mmu-miR-155-5p	*cebpβ*		[16, 35–37]
mmu-miR-190a-5p		*cebpα*	-
mmu-miR-190b-5p		*cebpα*	-
mmu-miR-205-5p		*cebpα*	[38]
mmu-miR-224-3p		*cebpα*	[39]
mmu-miR-27a-3p	*pparγ*	*pparγ*	[40, 41]
mmu-miR-27b-3p	*pparγ*	*pparγ*	[42–46]
mmu-miR-326-3p		*cebpα*	[47]
mmu-miR-329-3p	*cebpα*		-
mmu-miR-330-5p		*cebpα*	-
mmu-miR-362-3p	*cebpα*		-
mmu-miR-466f-5p		*cebpβ*	-
mmu-miR-466i-3p	*cebpα*		-
mmu-miR-466m-3p	*cebpα*		-
mmu-miR-466o-3p	*cebpα*		-
mmu-miR-671-5p		*cebpα*	-
mmu-miR-690	*cebpα*		-

CEBP α and β: Relative CCAAT enhancer-binding protein α and β; PPARγ: peroxisome proliferator-activated receptor γ.

### miRNA transfection

3T3-L1 pre-adipocytes at a confluence of approximately 80% were transfected with the DeliverX^TM^ Plus siRNA Transfection Kit (Affimetrix, Santa Clara, CA) following the manufacturer´s protocol with mirVana miRNA inhibitor mmu-miR-155-5p or mirVana miRNA inhibitor Negative Control (Applied Biosystems, Foster City, CA, USA). The final concentration of miRNA Inhibitors was established at 30 nM and the transfection period at 48 hours. These optimal conditions were determined in previous experiments carried out at 24, 48 and 72 hours in cells at different confluence statuses, and transfection efficiency was assessed using miRNA probes and fluorescent transfection controls.

At the same time, cells were stimulated to differentiate with DMEM containing 10% FCS, 10 μg/mL insulin, 0.5 mM isobutylmethylxanthine (IBMX), and 1 μM dexamethasone and treated with RSV at 25 μM or ethanol 95% (Control group) during 48 hours. Afterwards, the supernatant was removed and cells were used to RNA and protein extraction. Each experiment was performed 3 times.

### Extraction and analysis of RNA and quantification by Real Time reverse transcription-polymerase chain reaction (Real Time RT-PCR)

Total RNA sample containing small and large-size RNA from maturing pre-adipocytes was extracted with miRNeasy™ RNA isolation kit (Qiagen, Hilden, Germany) according to the manufacturer's protocol. Small-size RNA was used for miRNA expression analysis and large-size RNA to quantify the mRNA expression.

1.5 μg of large-size RNA of each sample was reverse-transcribed to first-strand complementary DNA (cDNA) using iScriptTM cDNA Synthesis Kit (Bio-Rad, Hercules, CA, USA). Sterol regulatory element binding transcription factor 1 (*srebf1*), krüppel-like factor 5 (*klf5*), liver x receptor α (*lxrα*) and cAMP responding element binding protein 1 (*creb1*) mRNA levels were quantified using Real-Time PCR with an iCyclerTM–MyiQTM Real-Time PCR Detection System (BioRad, Hercules, CA, USA) in the presence of SYBRGreen master mix (Applied Biosystems, Foster City, CA, USA). All sample mRNA levels were normalized to the values of 18S (Table 2).

**Table 2:** Primers for PCR amplification of each studied gene.

	Sense primer	Anti-sense primer
*srebf1*	5´- AAATCTTGCTGCCATTCG -3´	5´- TTGATCCCGGAAGCTCTGTG -3´
*klf5*	5´- CCGGAGACGATCTGAAACAC -3´	5´- GGAGCTGAGGGGTCAGATACTT -3´
*creb1*	5´- TTTGTCCTTGCTTTCCGAAT -3´	5´- CACTTTGGCTGGACATCTTG -3´
*lxrα*	5´- ATCGCCTTGCTGAAGACCTCTG -3´	5´- GATGGGGTTGATGAACTCCACC -3´
*18s*	5´- GTGGGCCTGCGGCTTAAT -3´	5´- GCCAGAGTCTCGTTCGTTATC -3´

Sterol regulatory element binding transcription factor 1 (*srebf1*); krüppel-like factor 5 (*klf5*); cAMP responding element binding protein 1 (*creb1*), liver x receptor α (*lxrα*); 18S ribosomal RNA (*18s*).

Reverse transcription of 10 ng of small-size RNA and PCR were performed with the TaqMan® MicroRNA Assay kit according to the manufacturer’s instructions (Applied Biosystems, Foster City, CA, USA). miRNA levels for miR-130b-3p, miR-155-5p, miR-27b-3p, miR-326-3p, miR-31-5p, miR-27a-3p, miR-144-3p, miR-205-5p and miR-224-3p were quantified using TaqMan® MicroRNA Assay (Applied Biosystems, Foster City, CA, USA) for each miRNA and normalized to the values of U6 snRNA. The miRNA assay sequences were as follows:

miR-130b-3p 5’- CAGUGCAAUGAUGAAAGGGCAU -3’
miR-155-5p 5’- UUAAUGCUAAUUGUGAUAGGGGU -3’
miR-27b-3p 5’- UUCACAGUGGCUAAGUUCUGC -3’
miR-326-3p 5’- CCUCUGGGCCCUUCCUCCAGU -3’
miR-31-5p 5’- AGGCAAGAUGCUGGCAUAGCUG -3’
miR-27a-3p 5’- UUCACAGUGGCUAAGUUCCGC—3’
miR-144-3p 5’- UACAGUAUAGAUGAUGUACU—3’
miR-205-5p 5’- UCCUUCAUUCCACCGGAGUCUG—3’
miR-224-3p 5’- AAAUGGUGCCCUAGUGACUACA—3’

All gene and miRNA expression results were expressed as fold changes of threshold cycle (Ct) value relative to controls using the 2^-ΔΔCt^ method [48].

After miRNA transfection assay, total RNA sample containing small and large-size RNA was extracted with miRNeasy™ RNA isolation kit (Qiagen, Hilden, Germany). Small-size RNA was used to mir-155 expression analysis and large-size RNA to quantify the mRNA expression of *cebpβ*. The expression levels of both mir-155 and *cebp*β were analyzed as explained before.

### Protein expression analysis

Total protein was isolated from maturing 3T3-L1 adipocytes using 150 μL of lysis buffer (2 nM tris-HCl, 0.1 M sodium chloride (NaCl), 1% Triton, 10% glycerol, 1 mM sodium orthovanadate (OvNa), 2 mM EDTA, 1 mM phenylmethylsulfonyl fluoride (PMSF), 2 mM sodium fluoride (FNa) and 1% protease inhibitor) and centrifuged (12.000g, 15 minutes, 4°C) to remove membranes and other proteic residues. Protein concentration was determined by BCA protein assay kit (Thermo Scientific, Wilmington, DE, USA). Total protein (20 μg) was subjected to 10% SDS-polyacrylamide gel, electroblotted onto PVDF membranes (Millipore, Bradford, MA, USA), and incubated with polyclonal rabbit anti-*cebpβ* (1:1000) and monoclonal mouse anti-tubulin (1:5000) (Santa-Cruz Biotech, CA, USA) overnight and afterwards with polyclonal goat anti-mouse IgG-HRP for *cebpβ* (1:5000) and polyclonal goat anti-rabbit for α-tubulin (1:5000) (Santa-Cruz Biotech, CA, USA) for 2 hours. Bound antibodies were visualized by an ECL system (Thermo Fisher Scientific Inc., Rockford, IL, USA) and quantified by Chemi-Doc MP imaging system (BioRad, CA, USA).

### Statistical analysis

Results are presented as mean ± standard error of the mean. Statistical analysis was performed using SPSS 24.0 (SPSS Inc. Chicago, IL, USA). Comparisons between each treatment and the controls were analyzed by Student’s *t* test. Statistical significance was set-up at the *p* < 0.05 level.

## Results and discussion

As stated in the introduction section, obesity is a real problem, and functional molecules may be a new effective tool for the management of this disease. Among them, resveratrol has been demonstrated as having beneficial effects in order to face obesity in both *in vitro* and *in vivo* models. Several published *in vitro* studies conclude that this polyphenol is able to inhibit the process of adipogenesis, leading to a lower amount of differentiated adipocytes and thus to a decrease in triglyceride accumulation [49–51]. Along the same lines, we previously demonstrated that resveratrol and its glucuronide and sulfate metabolites are able to block adipogenesis and to reduce triglyceride accumulation to the same extent in 3T3-L1 maturing pre-adipocytes [25].

Adipogenesis is a complex process governed by a tightly controlled network of transcription factors that coordinate a great number of genes [52–55]. At the centre of this network there are two principal adipogenic factors, *pparγ* and *cebpα*, whose expression is regulated by other transcription factors, such as *cebpβ* [27]. In recent years, miRNAs have been described as a potential group of adipogenic controllers. Indeed, a snapshot of miRNA profiling revealed a dramatic change of 21 miRNAs during 3T3-L1 adipocyte differentiation [56]. In this line, miR-155 and miR-27b have been shown to suppress the expression of *cebpβ* and *pparγ* in adipocytes. Therefore, these miRNAs could be considered one of the mechanisms by which the adipogenic process is inhibited [16, 35, 42].

Modulation of miRNA expression by dietary compounds is increasingly being investigated by scientists working in the field of functional ingredients and their potential capacity to prevent pathologies. Indeed, some dietary polyphenols, such as curcumin, epigallocatechin gallate or resveratrol have been demonstrated to suppress different cancer cells growth by up-regulating miRNAs [57]. Resveratrol has also been linked to modifications on miRNAs expression in heart myoblasts, which could explain its cardioprotective effect. Quercetin, coffee polyphenols and grape seed proanthocyanidins can target miR-122 in mice livers and control cholesterol and bile acid synthesis and fatty acid oxidation, and thus, prevent liver steatosis [58, 59]. With regard to regulation of adipogenesis through miRNAs, Zhu *et al*. demonstrated that epigallocatequines up-regulated the expression of miR-27a and miR-27b and down-regulated that of *pparγ* and *cebpα* [60]. The same effects were found by persimmon tannin treatment during adipogenesis [43]. By contrast, it seems that nonivamide, a capsaicin analogue, increases the expression of the miRNA mmu-let-7d-5p, which has been associated with decreased *pparγ* levels [61]. Other plant or fruit extracts have been also identified as adipogenic regulators via miRNAs [62, 63].

In view of all mentioned above, and considering that miRNAs can play a crucial role in the effect attributed to dietary polyphenols, in the present study we aimed to analyze the mechanisms by which RSV and its metabolites modified the gene expression of adipogenic regulators. For this purpose we focussed on the analysis of those potential miRNA (validated or predicted) targeting *pparγ*, *cebpβ* and *cebpα*, which were selected by using the miRWalk 2.0. database and a literature review.

**Fig 1:**
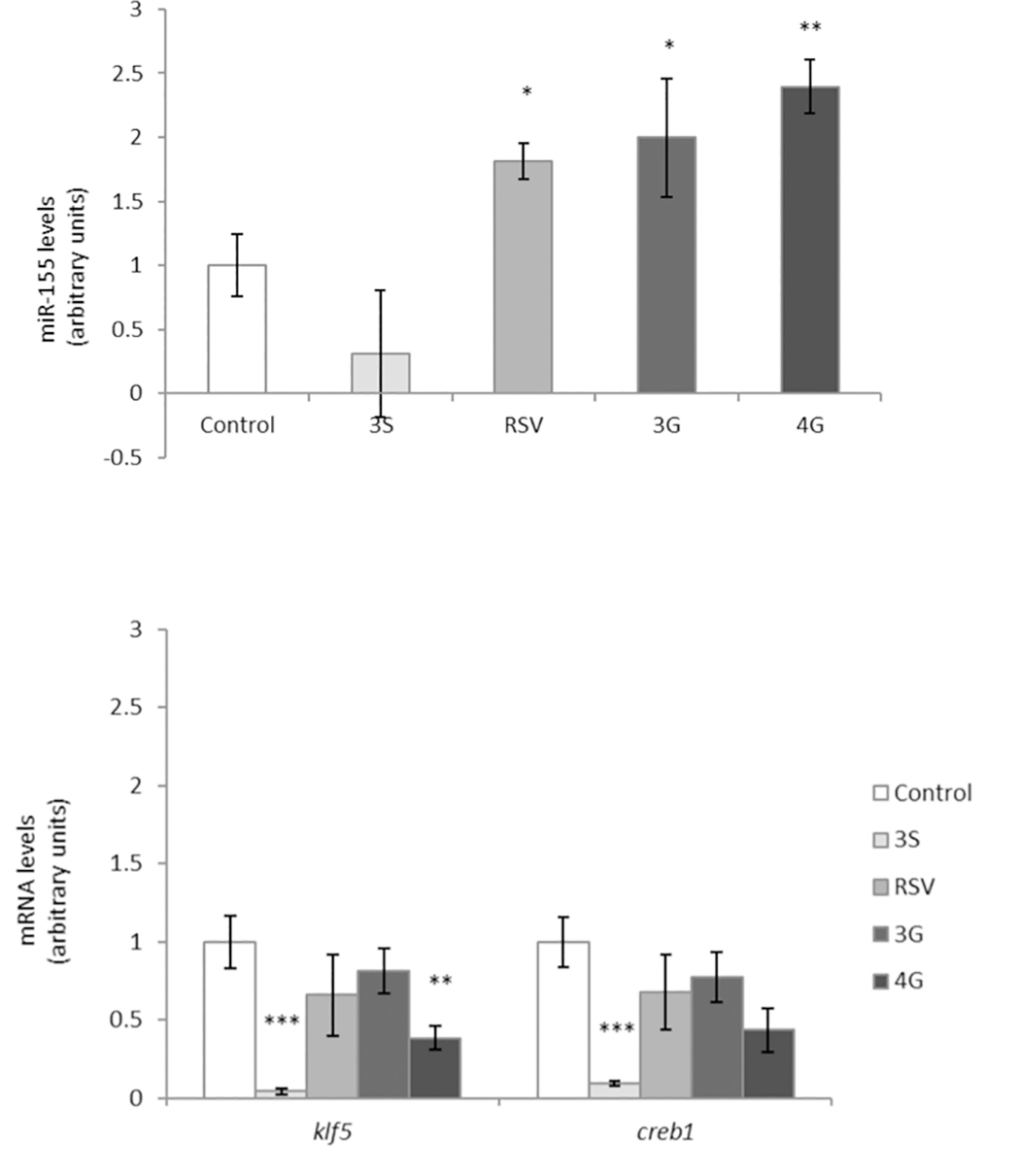
Effects of 25 μM of resveratrol (RSV) on the expression of mir-155 (A) and 25 μM of *trans*-resveratrol-3-O-sulfate (3S), *trans*-resveratrol-3-O-glucuronide (3G) and *trans*-resveratrol-4-O-glucuronide (4G) on *creb1* and *klf5* gene expression (B) in 3T3-L1 maturing pre-adipocytes treated from day 0 to day 8. Values are means ± SEM (Standard Error of the Mean) of three independent experiments carried out in sextuplicate. Comparisons between each treatment and the controls were analyzed by Student’s *t*-test. The asterisks represent differences versus the controls (*P < 0.05; **P < 0.01; ***P < 0.001).

**Fig 2:**
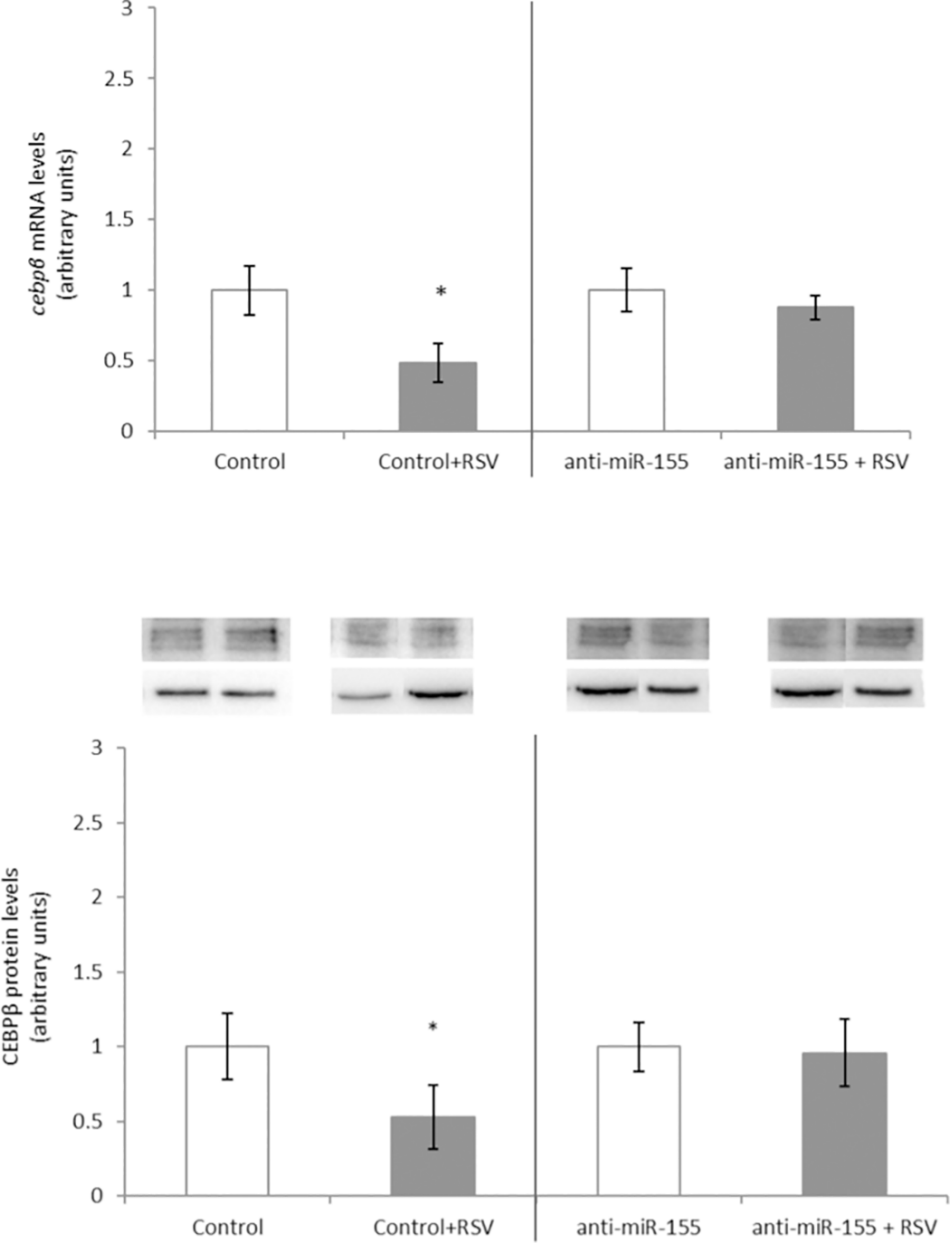
*cebpβ* gene (A) and protein (B) expressions after transfection with miR-155 inhibitor, with or without 25 μM RSV in 3T3-L1 maturing pre-adipocytes treated from day 0 to day 2. Values are means ± SEM (Standard Error of the Mean) of three independent experiments carried out in triplicate. Comparisons between each treatment and the controls were analyzed by Student’s *t*-test. The asterisks represent differences versus the controls (*P < 0.05).

MiR-155 and other genes that regulate the expression of *cebpβ* were measured (Table 1). RSV and the glucuronide metabolites increased miR-155 gene expression, but 3S metabolite did not (Fig 1A). These results could suggest that whereas RSV, 3G and 4G exert their effect via miR-155, 3S metabolite does not do so. In order to verify the mechanism of RSV and the glucuronide metabolites, maturing 3T3-L1 adipocytes were transfected with an anti-miR-155 compound while they were cultured in the presence or absence of RSV. After transfection, *cebpβ* gene and protein expression remained unchanged in treated cells (Fig 2), demonstrating that the polyphenol, and reportedly its glucuronide metabolites, inhibit the process of adipogenesis, at least in part, via miR-155. The modulation of miR-155 by RSV has been extensively studied in monocytes and macrophages. In these cells RSV was shown to increase miR-155 expression, to reduce the inflammatory response and to protect from atherosclerosis and hypertension [64–67]. Nevertheless, studies analyzing this regulatory pathway in adipocytes have not been carried out yet.

Taking into account that the sulfate metabolite did not exert any effect on miR-155 (Fig 1A), other regulatory routes that could lead to the reduction observed in *cebpβ* gene expression were analyzed. In the network of adipogenic transcription factors *creb1* plays a crucial role as *cebpβ* precursor [68–70]. Moreover, *klf5* is induced by *cebpβ/δ* and in turn controls *ppar*γ expression, thus mediating both the early and late stages of the differentiation program [71]. In the present study, 3S metabolite reduced gene expression of *creb1* and 3S and 4G that of *klf5* (Fig 1B). Therefore, it could be suggested that 3S metabolite orchestrated its effects on the initial phase of the adipogenesis in a transcriptional way, apparently without any influence of miRNA. By contrast, the 4G metabolite not only exerted its effect via miR-155, but also through *klf5*. The modulation of *cebpβ* was also observed by other polyphenols [72, 73].

**Fig 3:**
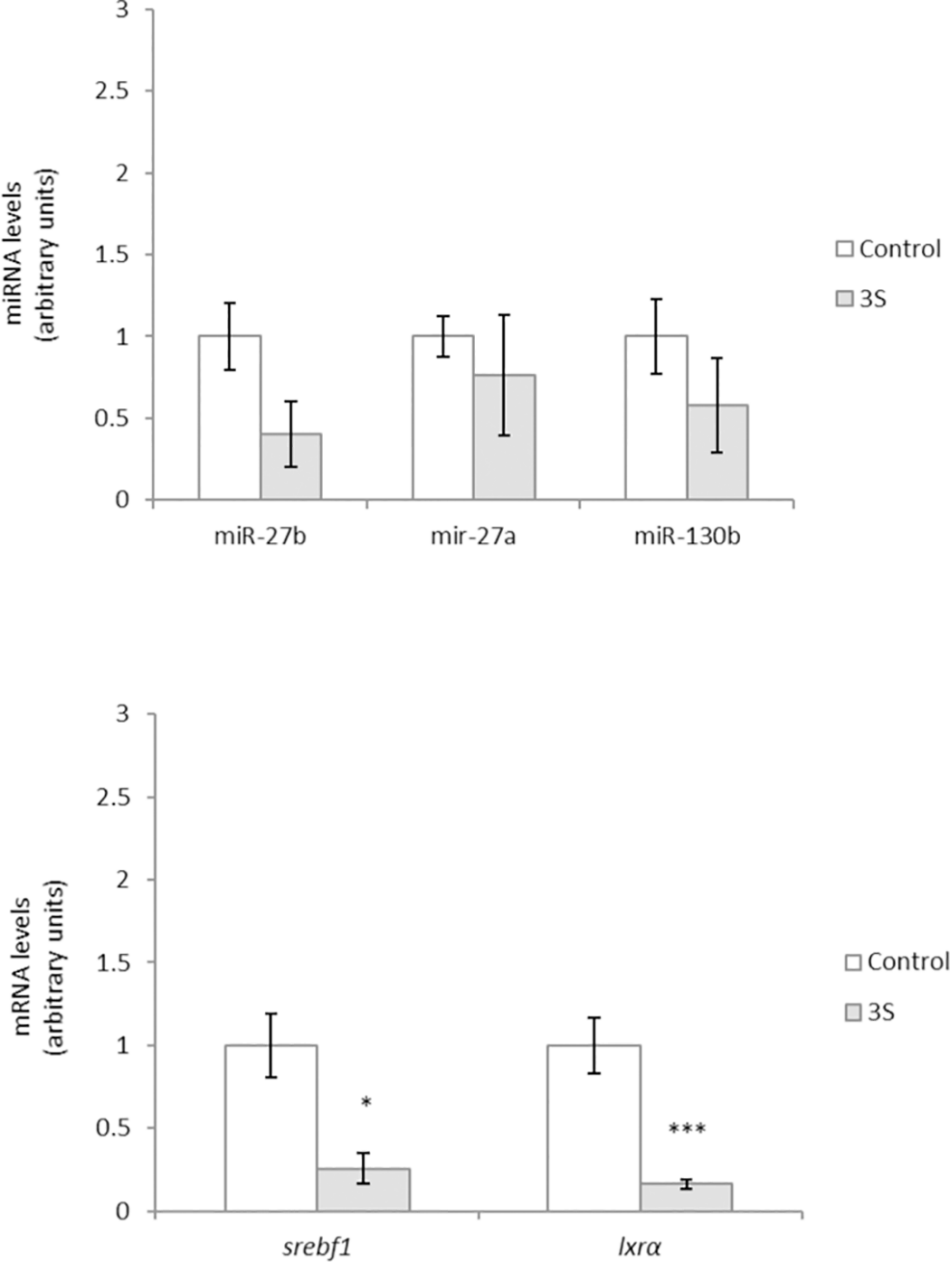
Effects of 25 μM of *trans*-resveratrol-3-*O*-sulfate (3S) on the expression of mir-27b, miR-27a and miR-130b (A) and on gene expression of *srebf1* and *lxrα* (B) in 3T3-L1 maturing pre-adipocytes treated from day 0 to day 8. Values are means ± SEM (Standard Error of the Mean) of three independent experiments carried out in sextuplicate. Comparisons between each treatment and the controls were analyzed by Student’s *t*-test. The asterisks represent differences versus the controls (*P < 0.05; ***P < 0.001).

MiR-27b, miR-27a and miR-130b were selected by miRWalk database (Table 1) as *pparγ* regulator. Sulfate metabolite did not change the expression of these miRNAs (Fig 3A). As in the case of *cebpβ*, other genes that are involved in the regulation of *pparγ* during adipogenesis (*srebf1* and *lxrα*) [74, 75] were analyzed. 3S metabolite reduced the expression of both genes (Fig 3B), which explains the changed induced by this metabolite in *pparγ* expression without changes in miR-27b, miR-27a and miR-130b. This fact was also observed with other anti-adipogenic pytochemicals such as apigenin [76], or black adzuki bean [77].

**Fig 4:**
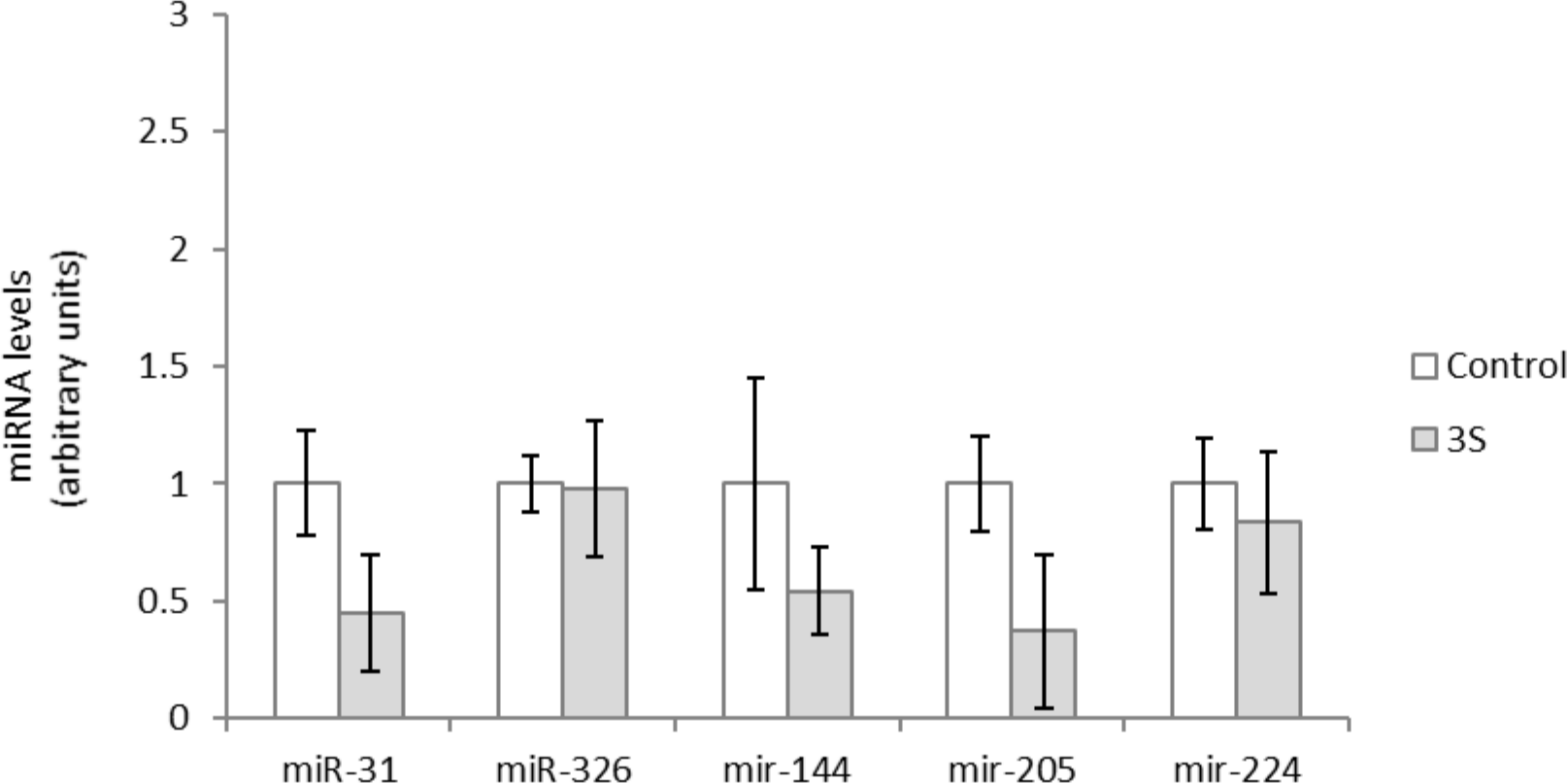
Effects of 25 μM of *trans*-resveratrol-3-*O*-sulfate (3S) on the expression of miR-326, miR-31, miR-144, miR-205 and miR-224 in 3T3-L1 maturing pre-adipocytes treated from day 0 to day 8. Values are means ± SEM (Standard Error of the Mean) of three independent experiments carried out in sextuplicates. Comparisons between each treatment and the controls were analyzed by Student’s *t*-test.

**Fig 5:**
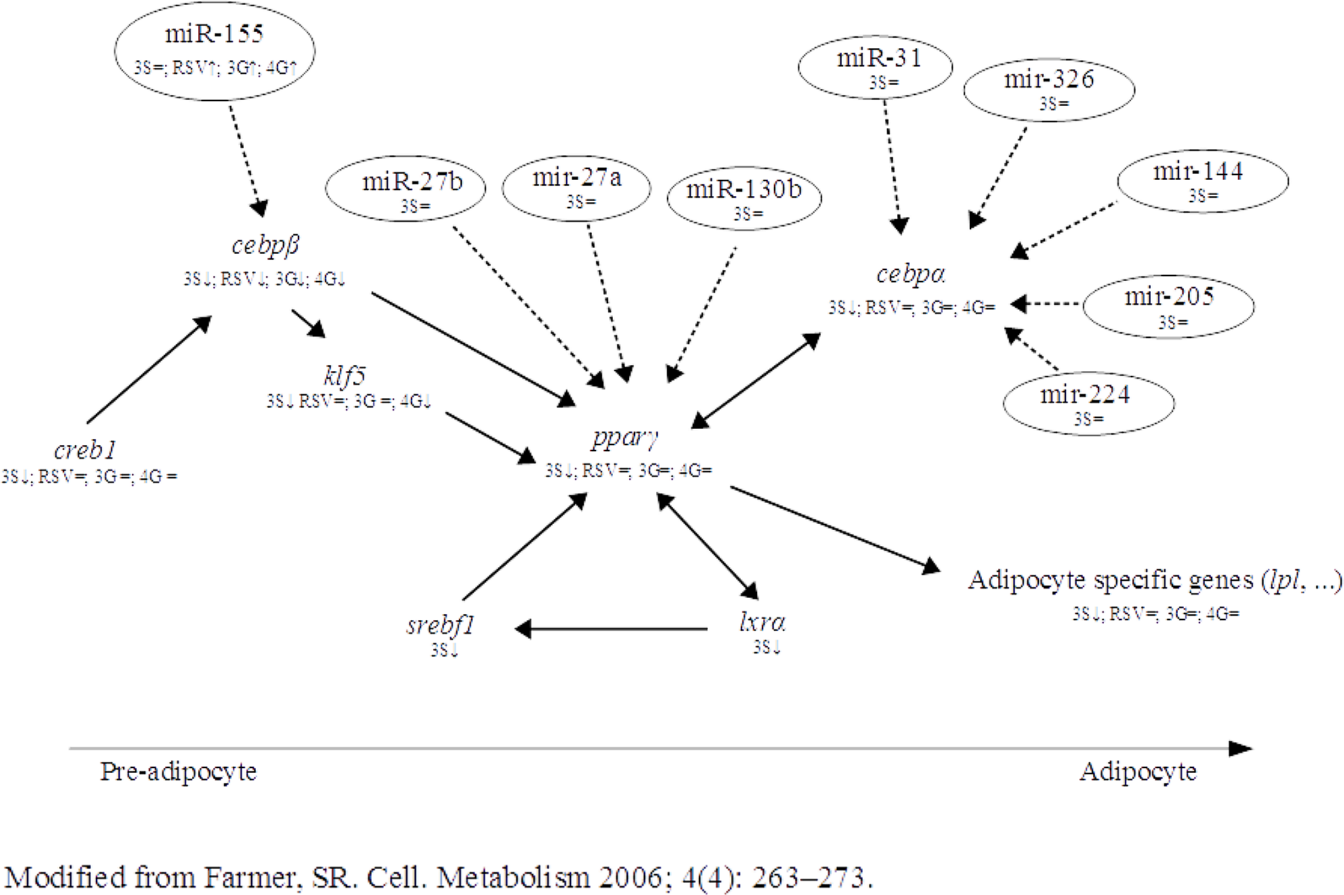
Genes and miRNAs involved in the inhibition of adipogenesis by resveratrol (RSV), *trans*-resveratrol-3-O-sulfate (3S), *trans*-resveratrol-3-O-glucuronide (3G) and *trans*-resveratrol-4-O-glucuronide (4G) in the pathways of the adipogenic process (modified from Farmer *et*. *al*. 2006).

Finally, we set out to analyze the miRNAs related to *cebpα*. For this purpose, miR-326, miR-31, miR-144, miR-205 and miR-224 were selected as miRNAs targeting *cebpα*, according to the computational analysis and literature (Table 1). None of these miRNAs were modified by 3S treatment (Fig 4), which suggests that its mechanism of action was not via miRNAs. The down-regulation observed by 3S on *pparγ*, can be considered itself one of the reasons for reduction in *cebpα*. These results, as a whole, suggest that 3S metabolite could exert its anti-adipogenic effect through adipogenic regulatory genes but not through miRNAs, as is the case of resveratrol and glucuronide metabolites (Fig 5).

This study presents the limitation that the experiments were performed in 3T3-L1 adipocytes. Therefore, data extrapolation to human is not completely possible. There are some differences in the metabolism and physiology of mouse and human adipogenesis such as differences in the modulation of *pparγ* [78]. However, the main species-specific differences in adipogenesis focus on when (and where) the products of the genes are made. However, the role of the master regulators is the same in both species, as far as we know. Taking into account the methodological difficulties that human adipocytes present for transfections and the heterogeneity of results in response to treatments, 3T3-L1 adipocytes were used in the present study.

## Conclusions

In summary, our study clearly suggests that the inhibitory effect on adipogenesis attributed to RSV and its glucuronide metabolites (3G and 4G) in 3T3-L1 adipocytes is mediated by the up-regulation of miR-155, which in turn leads to a down-regulation of *cebpβ* gene expression. In the case of 4G, *klf5* also contributed to this regulation. By contrast, the inhibitory effect observed in cells treated with 3S metabolite was not mediated via miRNAs. In this case, changes in *creb1*, *klf5*, *srebf1* and *lxrα*, explain the effects of this metabolite on adipogenesis.

## Abbreviations

RSV: *Trans*-resveratrol
3S: *trans*-resveratrol-3-*O*-sulfate
3G: *trans*-resveratrol-3-*O*-glucuronide
4G: *trans*-resveratrol-4’-*O*-glucuronide
miR-130b-3p: microRNA-130b-3p
miR-155-5p: microRNA-155-5p
miR-27b-3p: microRNA-27b-3p
miR-31-5p: microRNA-31-5p
miR-326-3p: microRNA-326-3p
miR-27a-3p: microRNA-27a-3p
miR-144-3p: microRNA-144-3p
miR-205-5p: microRNA-205-5p
miR-224-3p: microRNA-224-3p
